# Deferred Versus Upfront Cytoreductive Nephrectomy in MetaStatic Renal Cell Carcinoma: Comparative Survival Analysis in the Immunotherapy Era

**DOI:** 10.3390/cancers17193136

**Published:** 2025-09-26

**Authors:** Tao Xu, Paerhati Tuerxun, Ning Liu, Chencheng Ji, Kunlun Zhao, Yiguan Qian, Abudukelimu Abudushataer, Yang Li, Xiaotian Jiang, Zhongli Xiong, Min Wang, Ruipeng Jia, Yu-Zheng Ge

**Affiliations:** 1 General Clinical Research Center, Nanjing First Hospital, Nanjing Medical University, Nanjing 210012, China; 2 Department of Urology, Nanjing First Hospital, Nanjing Medical University, Nanjing 210006, China; 3 Department of Urology, Yining People’s Hospital, Yining 835099, China; 4 Department of Urology, People’s Hospital Campus of Yining General Hospital, Yining 835099, China

**Keywords:** metastatic renal cell carcinoma, cytoreductive nephrectomy, surgery timing, immunotherapy, population-based analysis

## Abstract

Background: The optimal timing of cytoreductive nephrectomy (CN) in metastatic renal cell carcinoma (mRCC) remains a subject of debate, particularly in the immunotherapy era. This study compares survival outcomes between deferred CN (dCN) and upfront CN (uCN) in mRCC patients receiving modern immunotherapy regimens in the real-world setting. Methods: We retrospectively analyzed the SEER database for mRCC patients diagnosed between 2016 and 2021 who underwent dCN or uCN. The primary endpoint was overall survival (OS), while the secondary endpoints were disease-specific survival (DSS) and other-cause specific survival (OCSS). Statistical analyses included propensity score matching (PSM), Kaplan–Meier survival curves, Cox proportional hazards modeling, as well as sensitivity, subgroup, and landmark analyses. Results: A total of 1892 mRCC patients were included, with 346 patients (18.3%) undergoing dCN and 1546 patients (81.7%) receiving uCN. Patients in the uCN group were characterized with lower T stage (*p* < 0.001), while those in the dCN group exhibited a higher incidence of lymph node involvement (*p* = 0.02) and sarcomatoid dedifferentiation (*p* = 0.002). Following 1:2 PSM, dCN demonstrated significantly better OS and DSS, but comparable OCSS to uCN. The sensitivity and subgroup analyses suggested that dCN may substantially improve the prognosis of mRCC patients across conditions. The landmark analysis showed that the survival advantage of dCN diminished after two years of follow-up. Conclusions: dCN may be associated with improved survival outcomes compared to uCN in selected mRCC patients receiving immunotherapy, and careful patient selection for dCN or uCN is essential.

## Simple Summary

The treatment landscape for metastatic renal cell carcinoma (mRCC) has undergone significant transformation, and cytoreductive nephrectomy (CN) persists as a viable intervention option in the immunotherapy era. However, the optimal timing of CN has not yet been clearly determined. In this large-scale, population-based, real-world study, we identified 1892 mRCC patients who underwent deferred CN (dCN) or upfront CN (uCN) from the SEER database. To capture contemporary nationwide treatment patterns, we included only patients diagnosed after 2016. Using propensity score matching, sensitivity, sub-group, and landmark analyses, we found that dCN was associated with superior survival compared to uCN in selected mRCC patients receiving immunotherapy, highlighting the importance of careful patient selection.

## 1. Introduction

Kidney cancer, primarily renal cell carcinoma (RCC), constitutes about 3% of all cancers [1]. Globally, around 434,840 new RCC cases and 155,953 RCC–related deaths occurred in 2022 [2]. Due to lack of typical clinical symptoms and early screening biomarkers, 20–30% of all RCC cases are diagnosed with metastatic RCC (mRCC) initially [3,4,5]. Despite significant advances in systemic therapy (ST), mRCC remains a formidable clinical challenge, with 5-year overall survival (OS) rates persistently below 20% [6,7].

Cytoreductive nephrectomy (CN) became the standard of care for mRCC patients in the cytokine therapy era after two randomized controlled trials (RCTs) demonstrated an OS benefit [8,9]. The therapeutic value of CN was subsequently challenged in the targeted therapy era, based on the equivocal findings from the CARMENA and SURTIME trials [10,11]. In 2015, the first immune checkpoint inhibitor (ICI; nivolumab) was approved for mRCC treatment following the CheckMate 025 trial [12], and the ST landscape for mRCC has evolved greatly with the approval of several different ICI regimens [13,14]. In the era of immunotherapy, the impact of CN on clinical outcomes continues to be investigated with ongoing RCTs [15,16,17], while some retrospective studies and post hoc analyses of previously reported trials have suggested potential benefits [18,19,20,21,22].

The optimal timing of CN continues to be debated in contemporary practice [23,24,25]. While upfront CN (uCN) before ST represented the historical standard, the cytokine era introduced the concept of deferred CN (dCN). The SURTIME trial evaluated these two strategies in sunitinib–treated mRCC patients, demonstrating a survival advantage for dCN compared to uCN [26]. However, in the current immunotherapy era, the comparative efficacy of dCN versus uCN remains inconclusive, primarily due to studies with limited statistical power and heterogeneous patient populations [20,27,28,29,30,31]. This knowledge gap underscores the critical need for robust real-world evidence to evaluate these strategies.

To optimize the clinical utility of CN in the modern era of immunotherapy, we comprehensively compared the clinical outcomes of dCN versus uCN with the largest reported cohort to date of 1892 newly diagnosed mRCC patients, according to real-world, population-based data.

## 2. Materials and Methods

### 2.1. Study Design

In this retrospective cohort study, we used data from the Surveillance, Epidemiology, and End Results (SEER) database (17 registries, November 2023 submission), which covers approximately 26.5% of the U.S. population [32]. Ethics approval and informed consent were waived because these publicly accessible data were de-identified [33,34]. The study complied with the Declaration of Helsinki and followed the Strengthening the Reporting of Observational Studies in Epidemiology (STROBE) reporting guidelines [35].

### 2.2. Patient Selection

**Figure 1:**
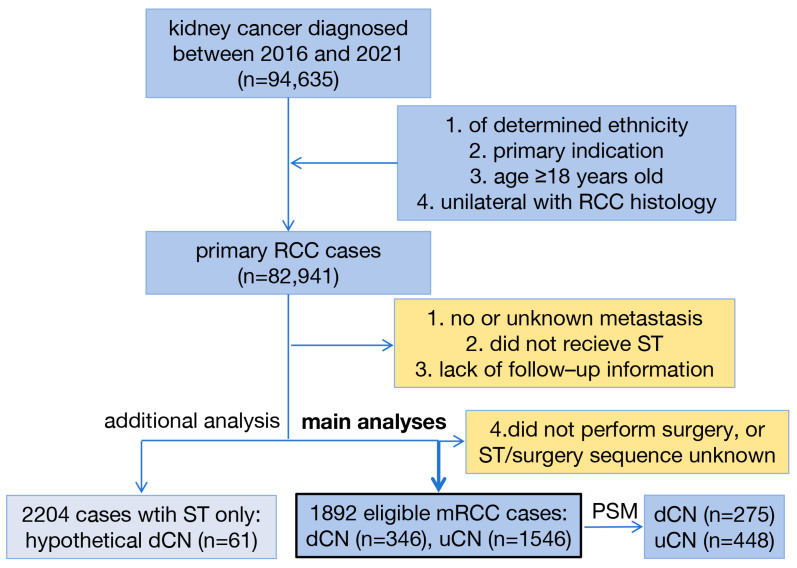
Study flowchart of patient screening. Out of 94,635 kidney cancer patients, 1892 eligible de novo mRCC cases were identified.

Consistent with our established methodology [34], we identified kidney cancer patients (site recode: kidney parenchyma, and ICD-O-3 codes: C64.9) using SEER Stat software (version 8.4.3). The study period (2016–2021) was selected to capture the immunotherapy era following the 2015 FDA approval of nivolumab for mRCC [36]. As outlined in Figure 1, the inclusion criteria consisted of: 1. specified ethnicity (White, Black, or other); 2. age of 18 years or older; 3. unilateral tumors with detailed RCC subtype classification, including clear cell RCC (ccRCC; 8310), non-clear cell RCC (nccRCC; 8260, 8290, 8311, 8316, 8317, 8319, 8323, 8480, and 8510), and RCC not otherwise specified (nosRCC; 8312) [37]; and 4. primary tumor designation. The exclusion criteria were set as: 1. unknown metastasis status based on the American Joint Committee on Cancer (AJCC) TNM classification, 2. absence of ST, 3. missing follow-up data, and 4. no surgery, or ST/surgery sequence unknown. In this study, uCN was defined as ST after surgery, while dCN as ST before surgery or ST before and after surgery, according to the database-defined data item: RX SUMM-Systemic/SurSeq.

### 2.3. Variables and Outcomes

We extracted the following variables from the SEER database: deidentified patient ID, age at diagnosis, sex, race/ethnicity, histological subtype, tumor grade, laterality, AJCC TNM stage, treatment details, malignancy history, and follow-up data [34]. The primary endpoint analyzed was OS, defined as the duration between initial diagnosis and death from any cause. Secondary endpoints included disease-specific survival (DSS) and other-cause-specific survival (OCSS), measured as the time from diagnosis to RCC-related death and non-RCC mortality, respectively.

### 2.4. Statistical Analysis

Descriptive statistics were generated using R package tableone (version 0.13.2) to summarize both continuous and categorical variables. Categorical variables were reported as absolute and relative frequencies, and compared using χ^2^ test or Fisher’s exact test, as appropriate. Continuous variables are presented as medians with interquartile ranges (IQRs), and those with nonnormal distribution were analyzed using the Kruskal–Wallis rank sum test. Survival curves were created using the Kaplan–Meier method and compared with the log-rank test, utilizing the R packages survminer (version 0.4.9) and survival (version 3.7-0). Additionally, the Cox proportional hazards model was employed to evaluate the differences in both primary and secondary endpoints, with results expressed as hazard ratios (HRs), 95% confidence intervals (CIs), and *p* values through the R package survival (version 3.7-0). Treatment effect heterogeneity across prespecified subgroups was assessed using interaction terms in the Cox proportional hazards model with the R package jstable (version 1.3.9).

To address potential observational bias, an exploratory sensitivity analysis was performed. According to the reported progressive disease (PD) rate in KEYNOTE-426 trial, it was estimated that approximately 15% of patients initially planned for dCN ultimately fail to undergo surgery due to PD [38]. These patients were randomly selected from the ST-only treatment group. To further minimize residual and selection bias, we performed PSM using the R package MatchIt (version 4.7.0) with a 2:1 nearest neighborhood matching ratio and a caliper width of 0.20. The propensity scores were generated using a logistic regression model that included all studied variables. The standardized mean difference (SMD) of baseline variables was calculated for both groups before and after matching, with an SMD < 0.1 considered balanced. Additionally, the landmark analyses were performed to assess clinical outcomes with the timepoint set at 2 years using R package jskm (version 0.5.11). All analyses were conducted using R software (version 4.3.1; R Foundation for Statistical Computing, Vienna, Austria), with a two–sided *p* value of 0.05 for statistical significance. Additional software details are provided in Supplementary Table S1.

## 3. Results

### 3.1. Baseline Characteristics

Between 2016 and 2021, a total of 94,635 kidney cancer patients were screened for eligibility, with 1892 synchronous mRCC patients included in this study (Figure 1). Among these patients, 346 patients (18.3%) underwent dCN, while 1546 patients (81.7%) received uCN. Baseline demographic and clinicopathological characteristics for all 1892 mRCC patients are summarized in Table 1. The median age at diagnosis was 62 years (IQR 55–69), with no significant difference between groups (*p* = 0.07). Patients in the uCN group were characterized with lower T stage (*p* < 0.001), while those in the dCN group exhibited a higher incidence of lymph node involvement (*p* = 0.02) and sarcomatoid dedifferentiation (*p* = 0.002). The other variables with respect to sex, race, histological subtype, laterality, radiation therapy, metastasis sites, and cancer history were well balanced between the two study groups.

### 3.2. Clinical Outcomes

**Figure 2:**
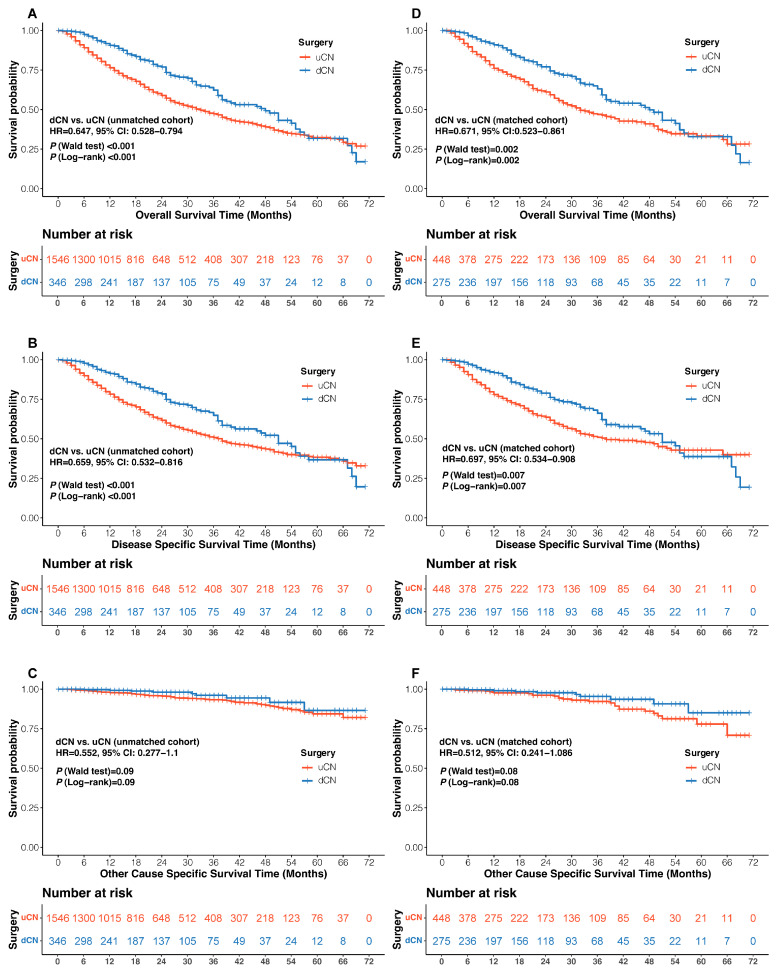
Kaplan–Meier plot of survival outcomes for metastatic renal cell carcinoma patients. Overall survival (**A**), Disease-specific survival (**B**), and Other-cause specific survival (**C**) in the unmatched cohort; Overall survival (**D**), Disease-specific survival (**E**), and Other-cause specific survival (**F**) in the matched cohort. mRCC, metastatic renal cell carcinoma; dCN, deferred cytoreductive nephrectomy; uCN, upfront cytoreductive nephrectomy; HR, hazard ratio; 95% CI, 95% confidence interval.

During a median follow-up of 19 months (IQR 9.00–32.75) for the dCN group and 19 months (8.00–37.00) for the uCN group, dCN presented a significant improvement in OS (Figure 2A; HR = 0.65, 95% CI: 0.53–0.79, *p* < 0.001) and DSS (Figure 2B; HR = 0.66, 95% CI: 0.53–0.82, *p* < 0.001) versus uCN. There was no significant difference in OCSS between the two groups (Figure 2C; HR = 0.55, 95% CI: 0.28–1.10, *p* = 0.09). To reduce the potential effect of confounding factors, the PSM method was applied (Figure S1), yielding a well-matched cohort of 723 patients (Table 1). Within the matched population, dCN remained associated with superior OS (Figure 2D; HR = 0.67, 95% CI: 0.52–0.86, *p* = 0.002) and DSS (Figure 2E; HR = 0.70, 95% CI: 0.53–0.91, *p* = 0.007), while maintaining comparable OCSS (Figure 2F; HR = 0.51, 95% CI: 0.24–1.09, *p* = 0.08) relative to uCN.

### 3.3. Sensitivity, Subgroup, and Landmark Analyses

In an exploratory sensitivity analysis, 61 additional mRCC patients were randomly selected from those receiving ST alone and were incorporated into the dCN group. As presented in Figure S2, a significant improved OS was observed (Figure S2A; HR = 0.81, 95% CI: 0.68–0.97, *p* = 0.02), while the DSS benefit approached statistical significance (Figure S2B; HR = 0.84, 95% CI: 0.69–1.01, *p* = 0.057).

**Figure 3:**
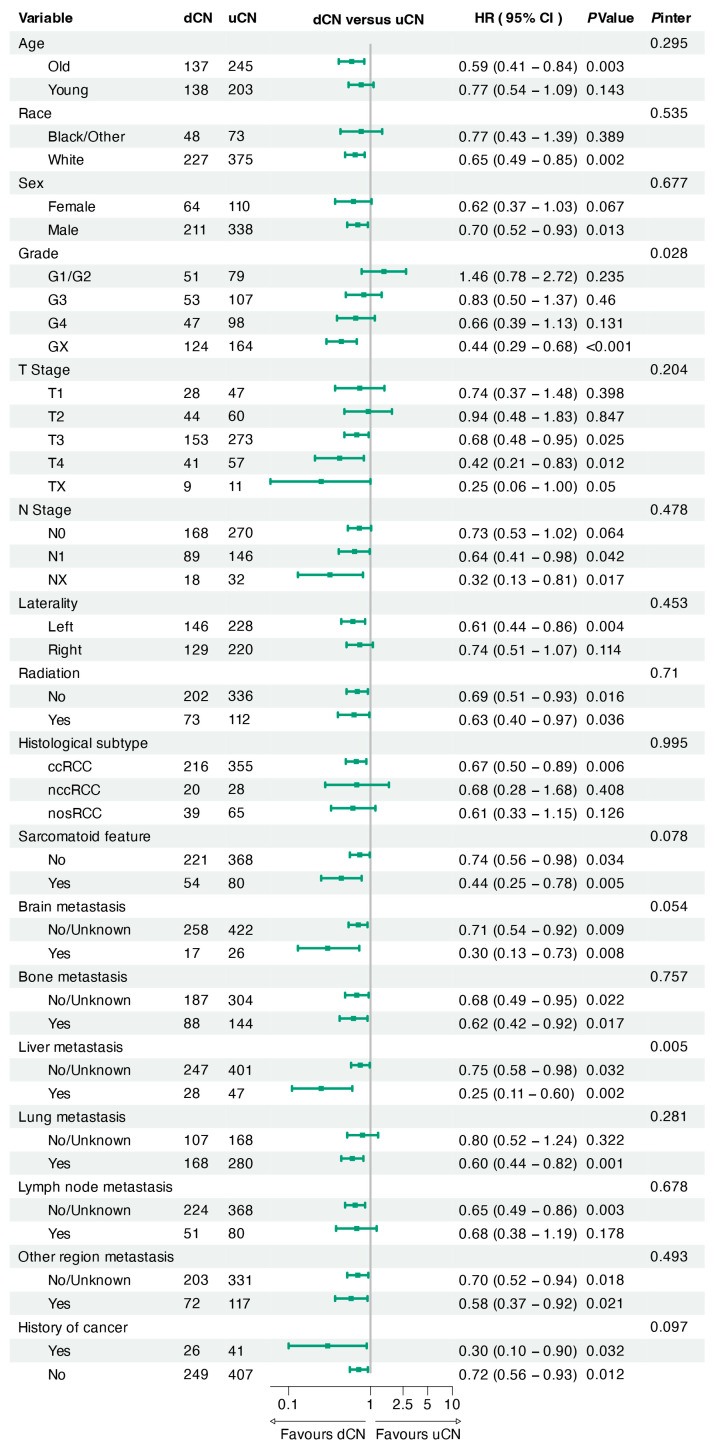
HR for the primary endpoint of overall survival across prespecified subgroups in the matched cohort. Old was defined as age > 60 years. dCN, deferred cytoreductive nephrectomy; uCN, upfront cytoreductive nephrectomy; ccRCC, clear cell renal cell carcinoma; nccRCC, non-clear cell renal cell carcinoma; nosRCC: renal cell carcinoma not otherwise specified; HR, hazard ratio; 95% CI, 95% confidence interval; *P*inter, *p* value for interaction.

In the further subgroup analysis focusing on the primary outcome (Figure 3), the HRs for OS were consistent across most subgroups, except liver metastasis status (*p* for interaction = 0.005). This finding was subsequently validated in the original cohort (Figure S3; *p* for interaction = 0.003). Given that ccRCC is the most prevalent histologic subtype of RCC, we performed a dedicated subgroup analysis after excluding other pathologic variants. In this homogeneous cohort, dCN maintained significantly survival benefits, demonstrating both improved OS (Figure S4A; HR = 0.69, 95% CI: 0.53–0.90, *p* = 0.006) and DSS (Figure S4B; HR = 0.72, 95% CI: 0.55–0.95, *p* = 0.02). Among mRCC patients with liver metastasis in the matched cohort, median OS was not reached (NR; IQR 35–NR) in the dCN group versus 15 months (IQR 8–32) in the uCN group (Figure S5A; HR = 0.26, 95% CI: 0.11–0.60, *p* = 0.002). This survival advantage was consistent in the original cohort (Figure S5B; HR = 0.29, 95% CI: 0.16–0.54, *p* < 0.001).

**Figure 4:**
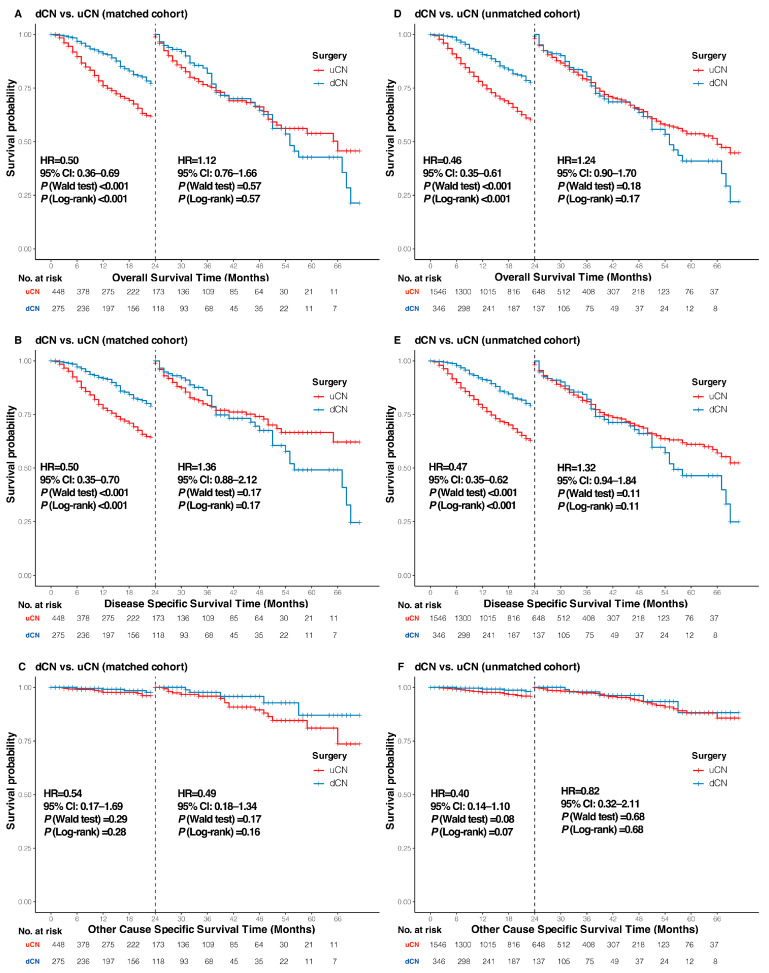
Landmark analysis of survival outcomes for metastatic renal cell carcinoma patients. Overall survival (**A**), Disease-specific survival (**B**), and Other-cause specific survival (**C**) in the matched cohort; Overall survival (**D**), Disease-specific survival (**E**), and Other-cause specific survival (**F**) in the unmatched cohort. mRCC, metastatic renal cell carcinoma; dCN, deferred cytoreductive nephrectomy; uCN, upfront cytoreductive nephrectomy; HR, hazard ratio; 95% CI, 95% confidence interval.

Due to the late crossing of the Kaplan–Meier curves for both OS and DSS in original and matched cohorts, landmark analyses were conducted to assess the clinical outcomes before and after the 2-year timepoint. In the matched cohort, OS (Figure 4A; HR = 0.50, 95% CI: 0.36–0.69, *p* < 0.001) and DSS (Figure 4B; HR = 0.50, 95% CI: 0.35–0.70, *p* < 0.001) were significantly superior in the dCN group compared with uCN within the first 2 years. After a two-year follow-up period, no significant difference emerged in OS (Figure 4A; HR = 1.12, 95% CI: 0.76–1.66, *p* = 0.57) or DSS (Figure 4B; HR = 1.36, 95% CI: 0.88–2.12, *p* = 0.17) between the two groups. These findings were replicated in the original cohort (Figure 4D,E), with OCSS remaining consistent in both cohorts across both time periods (Figure 4C,F).

## 4. Discussion

The treatment landscape for mRCC has evolved rapidly over the past three decades [14], with ongoing debate regarding the optimal use and timing of CN [23,39]. To our knowledge, we present the largest population-based study comparing the clinical outcomes of dCN and uCN in mRCC patients in the modern era of immunotherapy. Two major findings are as follows: 1. dCN demonstrated significant prognostic advantages over uCN in terms of both OS and DSS; 2. Proper patient selection is critical as the survival superiority of dCN diminished after two years of follow-up within the overall cohort, which remained in the lethal form with liver metastasis.

CN has historically served as the cornerstone of mRCC management in the cytokine era, with uCN emerging earlier than dCN [24,40]. The SURTIME trial was the first RCT to compare these two approaches in mRCC patients receiving Sunitinib, which demonstrated a significant OS benefit for dCN over uCN [11]. However, definitive conclusions were precluded due to the SURTIME trial’s poor accrual and use of outdated ST regimen. The optimal time for CN remains to be revisited in the contemporary immunotherapy era. Previous comparative studies have been constrained by limited sample size (ranged from 28 to 232), with most reporting no significant prognostic differences between dCN and uCN approaches [20,27,28,31]. In the present study, dCN was significantly associated with better OS (HR = 0.67, 95% CI: 0.52–0.86, *p* = 0.002 in matched cohort) and DSS (HR = 0.70, 95% CI: 0.53–0.91, *p* = 0.007 in matched cohort), which were in line with two recent meta-analyses of published studies [41,42].

Encouraged by existing evidence, three active RCTs, NORDIC-SUN (ClinicalTrials.gov identifier NCT03977571), PROBE (NCT04510597), and SEVURO-CN (NCT05753839), are investigating the role of CN in the ICI setting [15,16,17]. Among the three RCTs, two included dCN as the only form of CN [15,16]. However, uCN continues to be performed in a proportion of mRCC patients, underscoring the importance of strategic patient selection. The current study observed the late crossing of survival curves, consistent with the subgroup analyses of patients receiving ICI-based regimens [41,42]. The landmark analyses revealed a survival disparity during the first two years of follow-up, with no significant difference thereafter. This discrepancy may be attributable to enrollment bias, as long-term survivors (>2 years) predominantly comprised low-risk patients with favorable performance status. Furthermore, the subgroup based on liver metastasis found that dCN could substantially improve the prognosis of mRCC patients over uCN without survival curves crossing. Liver metastasis has been reported as one of the most lethal forms in mRCC, with a median progression-free survival of 5.5 months under immunotherapy [43]. Collectively, these findings emphasized the critical role of patient selection in choosing uCN or dCN. Although uCN is sometimes performed for symptom control in patients with gross hematuria and flank pain, it could be catastrophic for those prone to rapid progression receiving delayed ST due to uCN. Conversely, in the context of dCN, response to upfront ST can serve as a litmus test to identify the optimal CN candidates.

As an immunogenic malignancy, primary RCC harbors abundant neoantigens that prime the immune system through T-cell activation and clonal expansion, thereby enhancing tumor antigenicity [44]. The subsequent surgery could elicit a robust immune response, characterized by early interferon-γ and tumor necrosis factor production, which together form the biological rationale for dCN [23,39]. Furthermore, mRCC patients planned for dCN or uCN may become ineligible for subsequent CN or ST intervention due to the rapid disease progression or mortality. Notably, the time interval from ST to dCN is typically longer compared to that of uCN to subsequent ST, potentially introducing selection and observational biases which may inherently favor dCN over uCN [41].

In the absence of strong RCT evidence, our study had the strengths including the large-scale, population-based patient cohort and rigorous analysis methods. There are several limitations worth mentioning. First, the retrospective, observational design introduces potential biases (observational, selection, and attrition) that may reduce the reliability of the final conclusions. However, only one registered RCT (SEVURO-CN, NCT05753839) is comparing dCN versus uCN head-to-head for mRCC patients under immunotherapy, with estimated study completion by the end of 2031 [17]. Thus, several robust statistical methods, namely PSM, subgroup, landmark, and sensitivity analyses, have been adopted. Second, a wide array of immunotherapy regimens has been recommended for treating mRCC, and the inevitable gap between ideal recommendations and real-world clinical practice exists [13]. However, the SEER database lacks granular details on ST regimens, combinations, dosing, and sequencing. To optimize the accuracy and representativeness of nationwide treatment patterns in the current immunotherapy era, our analysis was restricted to patients diagnosed after 2016 [36], and the findings were in alignment with the meta-analyses of smaller studies across heterogeneous ST modalities [41,42]. Third, the absence of an exact time interval between ST and CN is a limitation. However, our findings are consistent with previous reports that utilized variably defined ST-CN intervals [29,30]. Additionally, two independent meta-analyses demonstrated consistent survival benefits with dCN across different interval thresholds, reinforcing its clinical validity [41,42]. Lastly, due to the structural design of the SEER database, certain detailed information including risk stratification metrics, tumor complexity assessments, and perioperative complications remains unavailable, potentially limiting analytical depth. Additionally, geographic coverage disparities, reporting variability, and data lag may influence the generalizability of our primary findings [34,45].

## 5. Conclusions

In summary, our analysis of a real-world, large-scale, population-based cohort revealed that dCN may be associated with superior survival compared with uCN in selected mRCC patients receiving immunotherapy, and careful patient selection for dCN or uCN is essential. The findings further highlighted the urgent need for validated predictive models to guide clinical practice in the modern immunotherapy era. In addition, adequately powered RCTs with prolonged follow-up are warranted to validate these observations across diverse ST regimens and risk strata. Such studies could elucidate the biological mechanisms underlying the differential clinical outcomes observed between dCN and uCN.

## Figures and Tables

**Table 1:** Baseline Characteristics of mRCC Patients in the Unmatched and Matched Population.

Variables	Before PSM			After PSM		
	dCN (n = 346)	uCN (n = 1546)	*p* Value	dCN (n = 275)	uCN (n = 448)	*p* Value
Age (years, median [IQR])	61.00 [54.00, 68.00]	62.00 [55.00, 69.00]	0.065	60.00 [54.00, 68.00]	61.00 [55.00, 68.00]	0.492
Race			0.151			0.921
Black	26 (7.5)	96 (6.2)		21 (7.6)	32 (7.1)	
Other	38 (11.0)	127 (8.2)		27 (9.8)	41 (9.2)	
White	282 (81.5)	1323 (85.6)		227 (82.5)	375 (83.7)	
Sex			0.06			0.763
Female	77 (22.3)	423 (27.4)		64 (23.3)	110 (24.6)	
Male	269 (77.7)	1123 (72.6)		211 (76.7)	338 (75.4)	
Grade			<0.001			0.148
G1	7 (2.0)	11 (0.7)		7 (2.5)	10 (2.2)	
G2	45 (13.0)	172 (11.1)		44 (16.0)	69 (15.4)	
G3	53 (15.3)	500 (32.3)		53 (19.3)	107 (23.9)	
G4	47 (13.6)	675 (43.7)		47 (17.1)	98 (21.9)	
GX	194 (56.1)	188 (12.2)		124 (45.1)	164 (36.6)	
T Stage			<0.001			0.619
T1	36 (10.4)	134 (8.7)		28 (10.2)	47 (10.5)	
T2	56 (16.2)	161 (10.4)		44 (16.0)	60 (13.4)	
T3	187 (54.0)	1070 (69.2)		153 (55.6)	273 (60.9)	
T4	57 (16.5)	161 (10.4)		41 (14.9)	57 (12.7)	
TX	10 (2.9)	20 (1.3)		9 (3.3)	11 (2.5)	
N Stage			0.02			0.947
N0	199 (57.5)	1010 (65.3)		168 (61.1)	270 (60.3)	
N1	125 (36.1)	445 (28.8)		89 (32.4)	146 (32.6)	
NX	22 (6.4)	91 (5.9)		18 (6.5)	32 (7.1)	
Laterality			0.65			0.619
Left	176 (50.9)	810 (52.4)		146 (53.1)	228 (50.9)	
Right	170 (49.1)	736 (47.6)		129 (46.9)	220 (49.1)	
Radiation Therapy			0.981			0.708
No	257 (74.3)	1152 (74.5)		202 (73.5)	336 (75.0)	
Yes	89 (25.7)	394 (25.5)		73 (26.5)	112 (25.0)	
Histology Subtype			0.2			0.864
ccRCC	273 (78.9)	1170 (75.7)		216 (78.5)	355 (79.2)	
nccRCC	22 (6.4)	144 (9.3)		20 (7.3)	28 (6.2)	
nosRCC	51 (14.7)	232 (15.0)		39 (14.2)	65 (14.5)	
Sarcomatoid Feature			0.002			0.618
No	287 (82.9)	1161 (75.1)		221 (80.4)	368 (82.1)	
Yes	59 (17.1)	385 (24.9)		54 (19.6)	80 (17.9)	
Bone Metastasis			0.909			0.995
No	237 (68.5)	1077 (69.7)		185 (67.3)	301 (67.2)	
Unknown	2 (0.6)	8 (0.5)		2 (0.7)	3 (0.7)	
Yes	107 (30.9)	461 (29.8)		88 (32.0)	144 (32.1)	
Brain Metastasis			0.674			0.965
No	321 (92.8)	1449 (93.7)		257 (93.5)	420 (93.8)	
Unknown	1 (0.3)	7 (0.5)		1 (0.4)	2 (0.4)	
Yes	24 (6.9)	90 (5.8)		17 (6.2)	26 (5.8)	
Liver Metastasis			0.392			0.738
No	302 (87.3)	1352 (87.5)		245 (89.1)	395 (88.2)	
Unknown	4 (1.2)	8 (0.5)		2 (0.7)	6 (1.3)	
Yes	40 (11.6)	186 (12.0)		28 (10.2)	47 (10.5)	
Lung Metastasis			0.366			0.664
No	133 (38.4)	538 (34.8)		105 (38.2)	162 (36.2)	
Unknown	2 (0.6)	15 (1.0)		2 (0.7)	6 (1.3)	
Yes	211 (61.0)	993 (64.2)		168 (61.1)	280 (62.5)	
Distant Lymph Node Metastasis			0.509			0.692
No	286 (82.7)	1256 (81.2)		223 (81.1)	364 (81.2)	
Unknown	1 (0.3)	13 (0.8)		1 (0.4)	4 (0.9)	
Yes	59 (17.1)	277 (17.9)		51 (18.5)	80 (17.9)	
Other Organ Metastasis			0.44			0.865
No	253 (73.1)	1157 (74.8)		202 (73.5)	328 (73.2)	
Unknown	1 (0.3)	12 (0.8)		1 (0.4)	3 (0.7)	
Yes	92 (26.6)	377 (24.4)		72 (26.2)	117 (26.1)	
History of Cancer			1			0.997
Yes	32 (9.2)	141 (9.1)		26 (9.5)	41 (9.2)	
No	314 (90.8)	1405 (90.9)		249 (90.5)	407 (90.8)	

mRCC, metastatic renal cell carcinoma; PSM, propensity score matching; dCN, deferred cytoreductive nephrectomy; uCN, upfront cytoreductive nephrectomy; IQR, interquartile range; ccRCC, clear cell renal cell carcinoma; nccRCC, non-clear cell renal cell carcinoma; nosRCC: renal cell carcinoma not otherwise specified.

## Data Availability

All data generated for this analysis were from the public SEER database, and available from the corresponding author on reasonable request.

## Supplementary Materials

The following supporting information can be downloaded at: https://www.mdpi.com/article/10.3390/cancers17193136/s1, Figure S1: Quality control of propensity score matching; Figure S2. Kaplan–Meier plot of survival outcomes for whole cohort of metastatic renal cell carcinoma patients; Figure S3. HR for the primary endpoint of overall survival across prespecified subgroups in the original cohort; Figure S4. Kaplan–Meier plot of survival outcomes for metastatic clear cell renal cell carcinoma patients; Figure S5. Kaplan–Meier plot of survival outcomes for renal cell carcinoma patients with liver metastasis. Table S1: Software resources listed in methods.

## References

[1] Siegel R.L., Kratzer T.B., Giaquinto A.N., Sung H., Jemal A. (2025). Cancer statistics, 2025. CA Cancer J. Clin..

[2] Filho A.M., Laversanne M., Ferlay J., Colombet M., Pineros M., Znaor A., Parkin D.M., Soerjomataram I., Bray F. (2025). The GLOBOCAN 2022 cancer estimates: Data sources, methods, and a snapshot of the cancer burden worldwide. Int. J. Cancer.

[3] Patel H.D., Gupta M., Joice G.A., Srivastava A., Alam R., Allaf M.E., Pierorazio P.M. (2019). Clinical Stage Migration and Survival for Renal Cell Carcinoma in the United States. Eur. Urol. Oncol..

[4] Young M., Jackson-Spence F., Beltran L., Day E., Suarez C., Bex A., Powles T., Szabados B. (2024). Renal cell carcinoma. Lancet.

[5] Netti G.S., De Luca F., Camporeale V., Khalid J., Leccese G., Troise D., Sanguedolce F., Stallone G., Ranieri E. (2025). Liquid Biopsy as a New Tool for Diagnosis and Monitoring in Renal Cell Carcinoma. Cancers.

[6] Singer E.A., Rumble R.B., Rathmell W.K., Van Veldhuizen P.J., Management of Metastatic Renal Clear Cell Cancer Guideline Expert Panel (2023). Management of Metastatic Renal Clear Cell Cancer: ASCO Guideline Rapid Recommendation Update. J. Clin. Oncol..

[7] Barragan-Carrillo R., Scavuzzo A., Jimenez-Rios M.A., Sobrevilla-Moreno N. (2025). Management of Metastatic Renal Cell Carcinoma: Guideline Updates. Eur. Urol. Focus..

[8] Flanigan R.C., Salmon S.E., Blumenstein B.A., Bearman S.I., Roy V., McGrath P.C., Caton J.R., Munshi N., Crawford E.D. (2001). Nephrectomy followed by interferon alfa-2b compared with interferon alfa-2b alone for metastatic renal-cell cancer. N. Engl. J. Med..

[9] Mickisch G.H., Garin A., van Poppel H., de Prijck L., Sylvester R., The European Organisation for Research and Treatment of Cancer (EORTC) Genitourinary Group (2001). Radical nephrectomy plus interferon-alfa-based immunotherapy compared with interferon alfa alone in metastatic renal-cell carcinoma: A randomised trial. Lancet.

[10] Mejean A., Ravaud A., Thezenas S., Colas S., Beauval J.B., Bensalah K., Geoffrois L., Thiery-Vuillemin A., Cormier L., Lang H. (2018). Sunitinib Alone or after Nephrectomy in Metastatic Renal-Cell Carcinoma. N. Engl. J. Med..

[11] Bex A., Mulders P., Jewett M., Wagstaff J., van Thienen J.V., Blank C.U., van Velthoven R., Del Pilar Laguna M., Wood L., van Melick H.H.E. (2019). Comparison of Immediate vs Deferred Cytoreductive Nephrectomy in Patients With Synchronous Metastatic Renal Cell Carcinoma Receiving Sunitinib: The SURTIME Randomized Clinical Trial. JAMA Oncol..

[12] Motzer R.J., Escudier B., McDermott D.F., George S., Hammers H.J., Srinivas S., Tykodi S.S., Sosman J.A., Procopio G., Plimack E.R. (2015). Nivolumab versus Everolimus in Advanced Renal-Cell Carcinoma. N. Engl. J. Med..

[13] Barragan-Carrillo R., Saad E., Saliby R.M., Sun M., Albiges L., Bex A., Heng D., Mejean A., Motzer R.J., Plimack E.R. (2025). First and Second-line Treatments in Metastatic Renal Cell Carcinoma. Eur. Urol..

[14] Shah N.J., Sura S., Shinde R., Shi J., Bupathi M., Vickery D., Perini R., Motzer R.J. (2025). Real-World Treatment Patterns and Clinical Outcomes Among Patients with Metastatic Renal Cell Carcinoma Post-Immune-Oncology and Vascular Endothelial Growth Factor Receptor Targeted Therapies. Cancers.

[15] Bell H., Cotta B.H., Salami S.S., Kim H., Vaishampayan U. (2022). “PROBE”ing the Role of Cytoreductive Nephrectomy in Advanced Renal Cancer. Kidney Cancer J..

[16] Iisager L., Ahrenfeldt J., Donskov F., Ljungberg B., Bex A., Lund L., Lyskjaer I., Fristrup N. (2024). Multicenter randomized trial of deferred cytoreductive nephrectomy in synchronous metastatic renal cell carcinoma receiving checkpoint inhibitors: The NORDIC-SUN-Trial. BMC Cancer.

[17] Park J.S., Kim J., Jeon J., Lee J., Jang W.S., Lee S.H., Han W.K., Choi Y.D., Koo K.C., Cho K.S. (2024). The role of cytoreductive nephrectomy in metastatic renal cell carcinoma in immune-oncology era (SEVURO-CN): Study protocol for a multi-center, prospective, randomized trial. Trials.

[18] Porta C., Bamias A., Zakopoulou R., Myint Z.W., Cavasin N., Iacovelli R., Pichler M., Kopecky J., Kucharz J., Rizzo M. (2023). Geographical differences in the management of metastatic de novo renal cell carcinoma in the era of immune-combinations. Minerva Urol. Nephrol..

[19] Bakouny Z., El Zarif T., Dudani S., Connor Wells J., Gan C.L., Donskov F., Shapiro J., Davis I.D., Parnis F., Ravi P. (2023). Upfront Cytoreductive Nephrectomy for Metastatic Renal Cell Carcinoma Treated with Immune Checkpoint Inhibitors or Targeted Therapy: An Observational Study from the International Metastatic Renal Cell Carcinoma Database Consortium. Eur. Urol..

[20] Takemura K., Ernst M.S., Navani V., Wells J.C., Bakouny Z., Donskov F., Basappa N.S., Wood L.A., Meza L., Pal S.K. (2024). Characterization of Patients with Metastatic Renal Cell Carcinoma Undergoing Deferred, Upfront, or No Cytoreductive Nephrectomy in the Era of Combination Immunotherapy: Results from the International Metastatic Renal Cell Carcinoma Database Consortium. Eur. Urol. Oncol..

[21] Grimm M.O., Oya M., Choueiri T.K., Motzer R.J., Schmidinger M., Quinn D.I., Gravis-Mescam G., Verzoni E., Van den Eertwegh A.J.M., di Pietro A. (2024). Impact of Prior Cytoreductive Nephrectomy on Efficacy in Patients with Synchronous Metastatic Renal Cell Carcinoma Treated with Avelumab plus Axitinib or Sunitinib: Post Hoc Analysis from the JAVELIN Renal 101 Phase 3 Trial. Eur. Urol..

[22] Makrakis D., Msaouel P., Karam J.A., Esagian S. (2024). Cytoreductive Nephrectomy in Metastatic Renal Cell Carcinoma Treated with Immune Checkpoint Inhibitors: A Systematic Review and Meta-analysis of Individual Patient Data. Eur. Urol. Focus..

[23] Das A., Shapiro D.D., Craig J.K., Abel E.J. (2023). Understanding and integrating cytoreductive nephrectomy with immune checkpoint inhibitors in the management of metastatic RCC. Nat. Rev. Urol..

[24] Monda S.M., Salami S.S., Vaishampayan U., Morgan T.M., Singhal U. (2025). Defining the Role of Surgical Resection in Metastatic Renal Cell Carcinoma: A Mini Review. Eur. Urol. Focus..

[25] Napolitano L., Manfredi C., Cirillo L., Fusco G.M., Passaro F., Abate M., La Rocca R., Mastrangelo F., Spirito L., Pandolfo S.D. (2023). Cytoreductive Nephrectomy and Metastatic Renal Cell Carcinoma: State of the Art and Future Perspectives. Medicina.

[26] Abu-Ghanem Y., van Thienen J.V., Blank C., Aarts M.J.B., Jewett M., de Jong I.J., Lattouf J.B., van Melick H.H.E., Wood L., Mulders P. (2022). Cytoreductive nephrectomy and exposure to sunitinib—A post hoc analysis of the Immediate Surgery or Surgery After Sunitinib Malate in Treating Patients With Metastatic Kidney Cancer (SURTIME) trial. BJU Int..

[27] Gross E.E., Li M., Yin M., Orcutt D., Hussey D., Trott E., Holt S.K., Dwyer E.R., Kramer J., Oliva K. (2023). A multicenter study assessing survival in patients with metastatic renal cell carcinoma receiving immune checkpoint inhibitor therapy with and without cytoreductive nephrectomy. Urol. Oncol..

[28] Yoshino M., Ishihara H., Nemoto Y., Nakamura K., Nishimura K., Tachibana H., Fukuda H., Toki D., Yoshida K., Kobayashi H. (2022). Therapeutic role of deferred cytoreductive nephrectomy in patients with metastatic renal cell carcinoma treated with nivolumab plus ipilimumab. Jpn. J. Clin. Oncol..

[29] Shen X.P., Xie M., Wang J.S., Guo X. (2023). Efficacy of immunotherapy-based immediate cytoreductive nephrectomy vs. deferred cytoreductive nephrectomy in metastatic renal cell carcinoma. Eur. Rev. Med. Pharmacol. Sci..

[30] Meagher M.F., Minervini A., Mir M.C., Cerrato C., Rebez G., Autorino R., Hampton L., Campi R., Kriegmair M., Linares E. (2024). Does the Timing of Cytoreductive Nephrectomy Impact Outcomes? Analysis of REMARCC Registry Data for Patients Receiving Tyrosine Kinase Inhibitor Versus Immune Checkpoint Inhibitor Therapy. Eur. Urol. Open Sci..

[31] Singla N., Hutchinson R.C., Ghandour R.A., Freifeld Y., Fang D., Sagalowsky A.I., Lotan Y., Bagrodia A., Margulis V., Hammers H.J. (2020). Improved survival after cytoreductive nephrectomy for metastatic renal cell carcinoma in the contemporary immunotherapy era: An analysis of the National Cancer Database. Urol. Oncol..

[32] Liu Y., Zhu K., Tian X., Chen P., Xiong Q., Li G., Ma X., Han R., Sun L., Shen Y. (2025). Individualized Prediction of Postoperative Survival in Gallbladder Cancer: A Nomogram Based on SEER Data and External Validation. Cancers.

[33] Leone J., Hassett M.J., Freedman R.A., Tolaney S.M., Graham N., Tayob N., Vallejo C.T., Winer E.P., Lin N.U., Leone J.P. (2024). Mortality Risks Over 20 Years in Men With Stage I to III Hormone Receptor-Positive Breast Cancer. JAMA Oncol..

[34] Liu N., Qian Y., Fan Y., Li Y., Ji C., Zhao K., Jiang X., Xiong Z., Wang M., Xu Z. (2025). Comparison of Partial Nephrectomy Versus Radical Nephrectomy for Metastatic Renal Cell Carcinoma in the Immunotherapy Era: A Propensity Score Matching, Population-Based Analysis. Ann. Surg. Oncol..

[35] von Elm E., Altman D.G., Egger M., Pocock S.J., Gotzsche P.C., Vandenbroucke J.P., Initiative S. (2007). The Strengthening the Reporting of Observational Studies in Epidemiology (STROBE) statement: Guidelines for reporting observational studies. Lancet.

[36] Adhikari A., Sapkota S., Gogia S., Kc O. (2024). Changes in the overall survival of patients with metastatic renal cell carcinoma in the era of immune-checkpoint inhibitors. Cancer Epidemiol..

[37] Moch H., Amin M.B., Berney D.M., Comperat E.M., Gill A.J., Hartmann A., Menon S., Raspollini M.R., Rubin M.A., Srigley J.R. (2022). The 2022 World Health Organization Classification of Tumours of the Urinary System and Male Genital Organs-Part A: Renal, Penile, and Testicular Tumours. Eur. Urol..

[38] Rini B.I., Plimack E.R., Stus V., Gafanov R., Hawkins R., Nosov D., Pouliot F., Alekseev B., Soulieres D., Melichar B. (2019). Pembrolizumab plus Axitinib versus Sunitinib for Advanced Renal-Cell Carcinoma. N. Engl. J. Med..

[39] Ingels A., Campi R., Capitanio U., Amparore D., Bertolo R., Carbonara U., Erdem S., Kara O., Klatte T., Kriegmair M.C. (2022). Complementary roles of surgery and systemic treatment in clear cell renal cell carcinoma. Nat. Rev. Urol..

[40] Leung D.K., Ko I.C., Siu B.W., Wong C.H., Yuen S.K., Ng C.F., Teoh J.Y. (2024). The Role of Surgery in Metastatic Renal Cell Carcinoma in 2024. Clin. Med. Insights Oncol..

[41] Esagian S.M., Karam J.A., Msaouel P., Makrakis D. (2025). Upfront Versus Deferred Cytoreductive Nephrectomy in Metastatic Renal Cell Carcinoma: A Systematic Review and Individual Patient Data Meta-analysis. Eur. Urol. Focus..

[42] Fong K.Y., Lim E.J., Wong H.C., Tay K.J., Ho H.S.S., Yuen J.S.P., Aslim E., Chen K., Gan V.H.L. (2025). Deferred cytoreductive nephrectomy in patients with metastatic renal cell carcinoma: A systematic review and patient-level meta-analysis. Urol. Oncol..

[43] Rini B.I., Powles T., Atkins M.B., Escudier B., McDermott D.F., Suarez C., Bracarda S., Stadler W.M., Donskov F., Lee J.L. (2019). Atezolizumab plus bevacizumab versus sunitinib in patients with previously untreated metastatic renal cell carcinoma (IMmotion151): A multicentre, open-label, phase 3, randomised controlled trial. Lancet.

[44] Pauken K.E., Alhalabi O., Goswami S., Sharma P. (2025). Neoadjuvant immune checkpoint therapy: Enabling insights into fundamental human immunology and clinical benefit. Cancer Cell.

[45] Jiang Y., Wang P., Tian Y. (2025). The Impact of Neoadjuvant Chemotherapy on Survival Outcomes in Gastric Signet-Ring Cell Carcinoma: An International Multicenter Study. Cancers.

