# Supplementary material for: Deferred Versus Upfront Cytoreductive Nephrectomy in MetaStatic Renal Cell Carcinoma: Comparative Survival Analysis in the Immunotherapy Era
Source: Cancers (Basel). 2025 Sep 26;17(19):3136. doi: 10.3390/cancers17193136 (PMC12523447; doi:10.3390/cancers17193136)
Supplement: Supplementary file 1 [file cancers-17-03136-s001.zip › cancers-3766960-supplementary.pdf]

Deferred versus upfront cytoreductive nephrectomy in metastatic renal cell carcinoma: survival outcomes in the immunotherapy era

Tao Xu[1,2*], Paerhati Tuerxun[3,4*], Ning Liu[2], Chencheng Ji[2], Kunlun Zhao[3,4], Yiguan Qian[2], Abudukelimu Abudushataer[3,4], Yang Li[2], Xiaotian Jiang[2], Zhongli Xiong[2], Min Wang[2], Ruipeng Jia[2,] Yu-Zheng Ge[2,3,4¶]

1. General Clinical Research Center, Nanjing First Hospital, Nanjing Medical University, Nanjing, Jiangsu, People's Republic of China
2. Department of Urology, Nanjing First Hospital, Nanjing Medical University, Nanjing, Jiangsu, People's Republic of China
3. Department of Urology, Yining People's Hospital, Yining, Xinjiang, People's Republic of China
4. Department of Urology, People's Hospital Campus of Yining General Hospital, Yining, Xinjiang, People's Republic of China

* These two authors (Tao Xu and Paerhati Tuerxun) contributed equally to this study.

¶ Correspondence:
Yu-Zheng Ge, MD, PhD
Department of Urology,
Associate Professor of Urology,
Deputy Director of Department of Urology,
Nanjing First Hospital, Nanjing Medical University,
E-mail: geyuzheng@njmu.edu.cn

Table S1. Software resources listed in methods

Software resources	Version	Uniform Resource Locator
tableone	0.13.2	https://github.com/kaz-yos/tableone
survminer	0.4.9	https://github.com/kassambara/survminer
survival	3.7-0	https://github.com/therneau/survival
jstable	1.3.9	https://github.com/jinseob2kim/jstable
MatchIt	4.7.0	https://github.com/kosukeimai/MatchIt
jskm	0.5.11	https://github.com/jinseob2kim/jskm
R software	4.3.1	https://www.r-project.org/

Legends of Supplementary Figures:

Figure S1. Quality control of propensity score matching. The propensity score distribution of both groups with jitter plot (A) and histogram (B); the LOVE plot of standardized mean difference (C).

Figure S2. Kaplan–Meier plot of survival outcomes for whole cohort of metastatic renal cell carcinoma patients. Overall survival (A) and disease specific survival (B) in the whole cohort. dCN, deferred cytoreductive nephrectomy; uCN, upfront cytoreductive nephrectomy; HR, hazard ratio; 95% CI, 95% confidence interval.

Figure S3. HR for the primary endpoint of overall survival across prespecified subgroups in the original cohort. Old was defined as age >60 years. dCN, deferred cytoreductive nephrectomy; uCN, upfront cytoreductive nephrectomy; ccRCC, clear cell renal cell carcinoma; nccRCC, non-clear cell renal cell carcinoma; nosRCC: renal cell carcinoma not otherwise specified; HR, hazard ratio; 95% CI, 95% confidence interval; *P*inter, *P* value for interaction.

Figure S4. Kaplan–Meier plot of survival outcomes for metastatic clear cell renal cell carcinoma patients. Overall survival (A) and disease specific survival (B) in the orignial cohort. dCN, deferred cytoreductive nephrectomy; uCN, upfront cytoreductive nephrectomy; HR, hazard ratio; 95% CI, 95% confidence interval.

Figure S5. Kaplan–Meier plot of survival outcomes for renal cell carcinoma patients with liver metastasis. Overall survival in the matched cohort (A) and unmatched cohort (B). mRCC, metastatic renal cell carcinoma; dCN, deferred cytoreductive nephrectomy; uCN, upfront cytoreductive nephrectomy; HR, hazard ratio; 95% CI, 95% confidence interval.

Figure S1

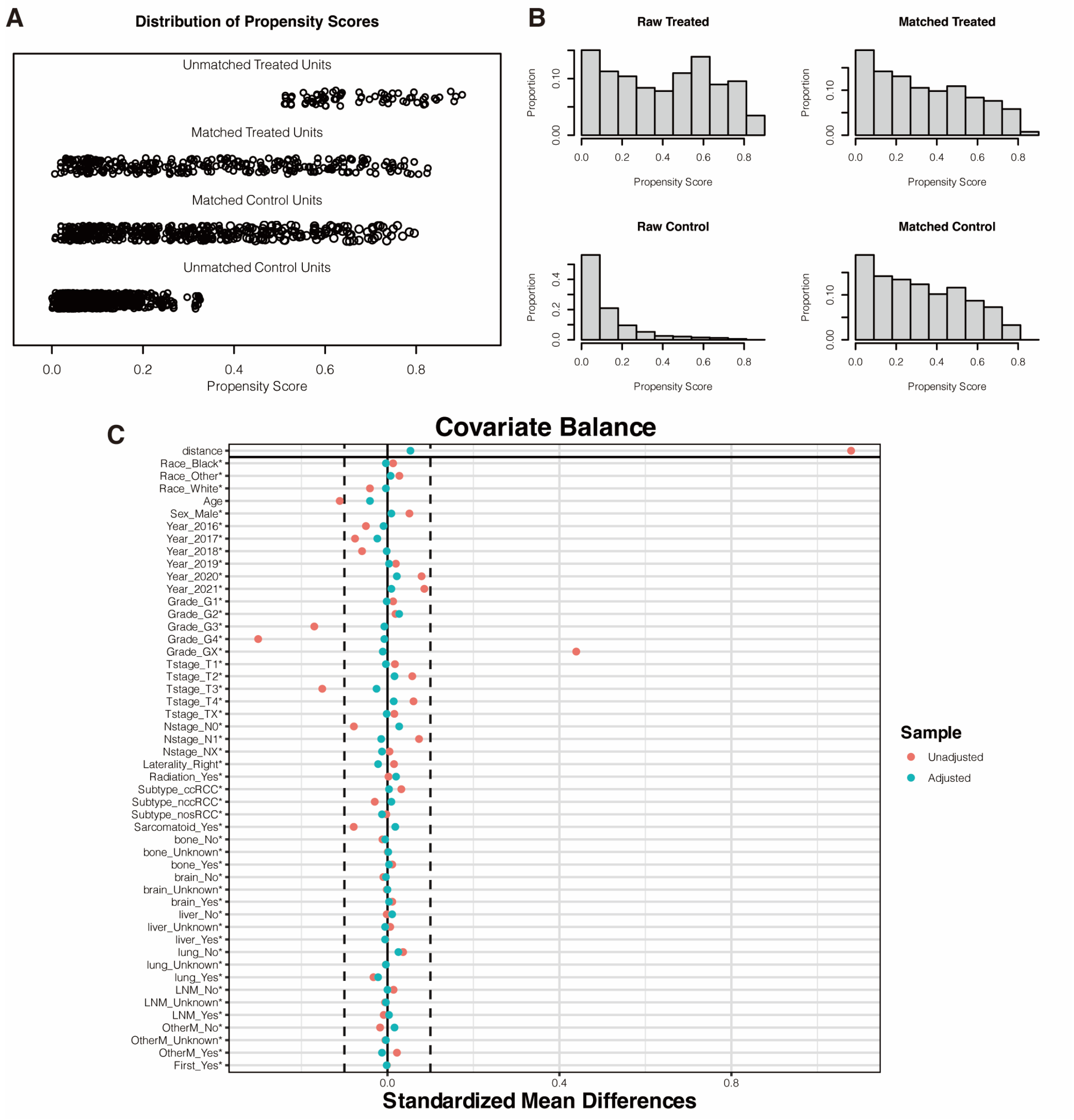

A
Distribution of Propensity Scores
Unmatched Treated Units
Matched Treated Units
Matched Control Units
Unmatched Control Units
Propensity Score
B
Raw Treated
Matched Treated
Raw Control
Matched Control
Proportion
Propensity Score
C
Covariate Balance
Standardized Mean Differences
Sample
Unadjusted
Adjusted
distance
Race_Black*
Race_Other*
Race_White*
Age
Sex_Male*
Year_2016*
Year_2017*
Year_2018*
Year_2019*
Year_2020*
Year_2021*
Grade_G1*
Grade_G2*
Grade_G3*
Grade_G4*
Grade_GX*
Tstage_T1*
Tstage_T2*
Tstage_T3*
Tstage_T4*
Tstage_TX*
Nstage_N0*
Nstage_N1*
Nstage_NX*
Laterality_Right*
Radiation_Yes*
Subtype_ccRCC*
Subtype_nccRCC*
Subtype_nosRCC*
Sarcomatoid_Yes*
bone_No*
bone_Unknown*
bone_Yes*
brain_No*
brain_Unknown*
brain_Yes*
liver_No*
liver_Unknown*
liver_Yes*
lung_No*
lung_Unknown*
lung_Yes*
LNM_No*
LNM_Unknown*
LNM_Yes*
OtherM_No*
OtherM_Unknown*
OtherM_Yes*
First_Yes*

Figure S2

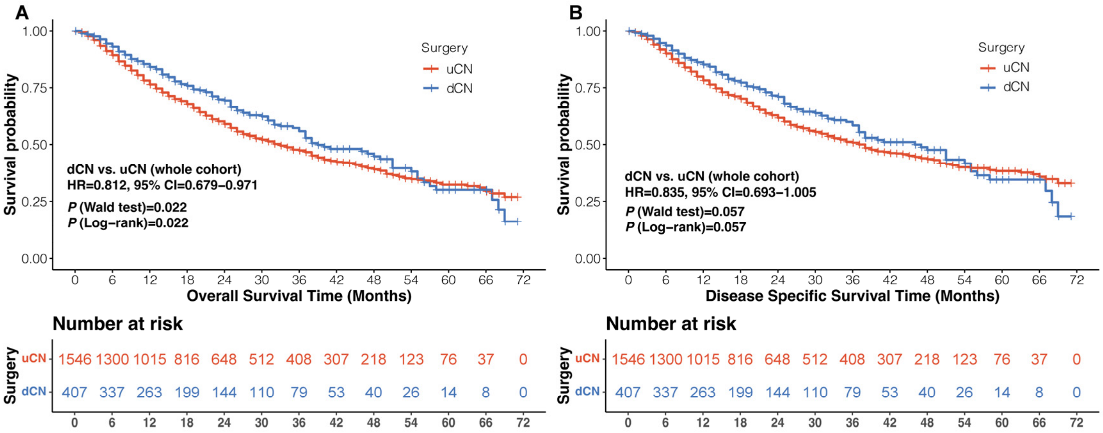
A
B
Survival probability
Surgery
uCN
dCN
dCN vs. uCN (whole cohort)
HR=0.812, 95% CI=0.679–0.971
P (Wald test)=0.022
P (Log−rank)=0.022
Overall Survival Time (Months)
dCN vs. uCN (whole cohort)
HR=0.835, 95% CI=0.693–1.005
P (Wald test)=0.057
P (Log−rank)=0.057
Disease Specific Survival Time (Months)
Number at risk
uCN 1546 1300 1015 816 648 512 408 307 218 123 76 37 0
dCN 407 337 263 199 144 110 79 53 40 26 14 8 0
0 6 12 18 24 30 36 42 48 54 60 66 72

Figure S3

Variable	dCN	uCN	dCN versus uCN	HR (95% CI)	*P* Value	*P* inter
Age						0.393
Old	177	887		0.59 (0.44 – 0.79)	<0.001	
Young	169	659		0.71 (0.53 – 0.94)	0.016	
Race						0.629
Black/Other	64	223		0.58 (0.35 – 0.95)	0.03	
White	282	1323		0.66 (0.53 – 0.83)	<0.001	
Sex						0.596
Female	77	423		0.59 (0.39 – 0.90)	0.014	
Male	269	1123		0.68 (0.54 – 0.86)	0.001	
Grade						0.024
G1/G2	52	183		1.12 (0.66 – 1.89)	0.675	
G3	53	500		0.91 (0.60 – 1.38)	0.647	
G4	47	675		0.77 (0.48 – 1.22)	0.267	
GX	194	188		0.46 (0.32 – 0.65)	<0.001	
T Stage						0.514
T1	36	134		0.83 (0.48 – 1.42)	0.492	
T2	56	161		0.76 (0.43 – 1.34)	0.341	
T3	187	1070		0.63 (0.48 – 0.84)	0.002	
T4	57	161		0.46 (0.27 – 0.78)	0.004	
TX	10	20		0.52 (0.18 – 1.50)	0.227	
N Stage						0.125
N0	199	1010		0.75 (0.57 – 0.99)	0.04	
N1	125	445		0.48 (0.35 – 0.67)	<0.001	
NX	22	91		0.49 (0.23 – 1.03)	0.059	
Laterality						0.551
Left	176	810		0.68 (0.52 – 0.90)	0.008	
Right	170	736		0.61 (0.45 – 0.82)	0.001	
Radiation						0.671
No	257	1152		0.66 (0.52 – 0.85)	0.001	
Yes	89	394		0.60 (0.41 – 0.87)	0.007	
Histological subtype						0.735
ccRCC	273	1170		0.66 (0.52 – 0.84)	0.001	
nccRCC	22	144		0.54 (0.26 – 1.11)	0.094	
nosRCC	51	232		0.74 (0.44 – 1.23)	0.247	
Sarcomatoid feature						0.481
No	287	1161		0.69 (0.55 – 0.86)	0.001	
Yes	59	385		0.57 (0.35 – 0.91)	0.018	
Brain metastasis						0.351
No/Unknown	322	1456		0.66 (0.53 – 0.82)	<0.001	
Yes	24	90		0.46 (0.23 – 0.93)	0.03	
Bone metastasis						0.713
No/Unknown	239	1085		0.62 (0.47 – 0.81)	<0.001	
Yes	107	461		0.66 (0.48 – 0.91)	0.011	
Liver metastasis						0.003
No/Unknown	306	1360		0.73 (0.59 – 0.91)	0.005	
Yes	40	186		0.29 (0.16 – 0.54)	<0.001	
Lung metastasis						0.826
No/Unknown	135	553		0.67 (0.47 – 0.95)	0.024	
Yes	211	993		0.64 (0.50 – 0.82)	0.001	
Lymph node metastasis						0.98
No/Unknown	287	1269		0.65 (0.52 – 0.81)	<0.001	
Yes	59	277		0.64 (0.41 – 1.01)	0.057	
Other region metastasis						0.719
No/Unknown	254	1169		0.66 (0.52 – 0.84)	0.001	
Yes	92	377		0.62 (0.42 – 0.91)	0.015	
History of cancer						0.033
Yes	32	141		0.23 (0.08 – 0.63)	0.004	
No	314	1405		0.70 (0.57 – 0.86)	0.001	

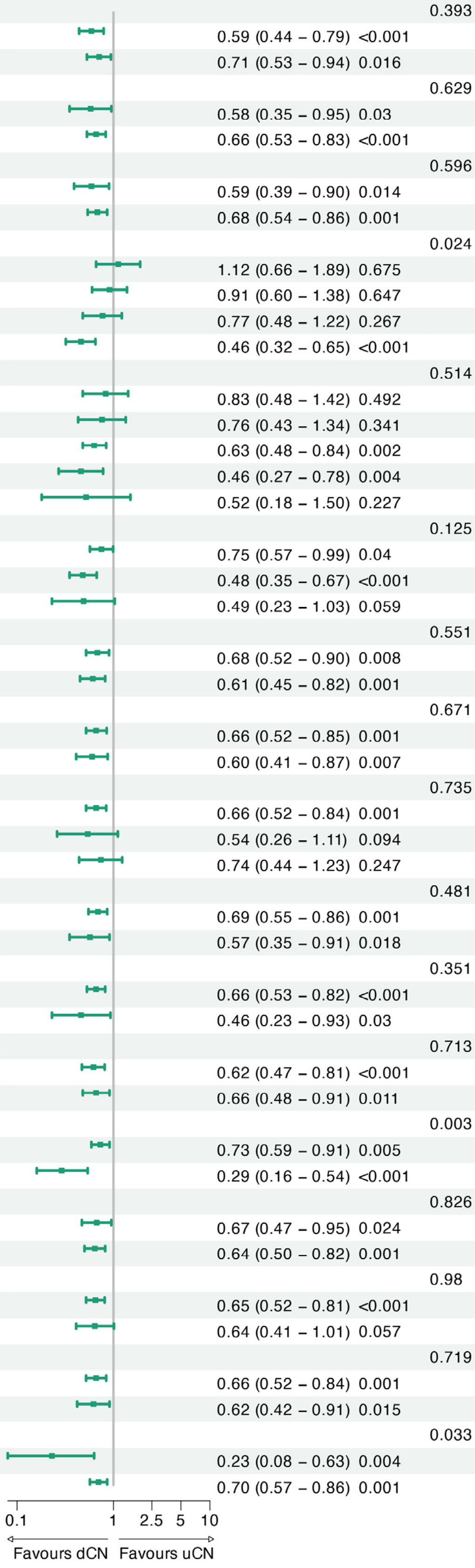

Figure S4

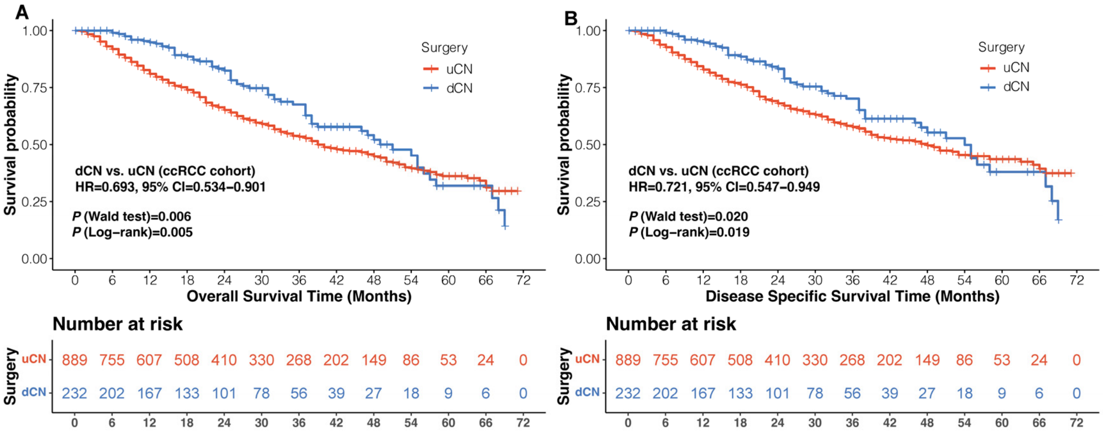
A
B
Survival probability
1.00
0.75
0.50
0.25
0.00
Surgery
uCN
dCN
dCN vs. uCN (ccRCC cohort)
HR=0.693, 95% CI=0.534–0.901
P (Wald test)=0.006
P (Log−rank)=0.005
Overall Survival Time (Months)
dCN vs. uCN (ccRCC cohort)
HR=0.721, 95% CI=0.547–0.949
P (Wald test)=0.020
P (Log−rank)=0.019
Disease Specific Survival Time (Months)
0 6 12 18 24 30 36 42 48 54 60 66 72
Number at risk
uCN 889 755 607 508 410 330 268 202 149 86 53 24 0
dCN 232 202 167 133 101 78 56 39 27 18 9 6 0

Figure S5

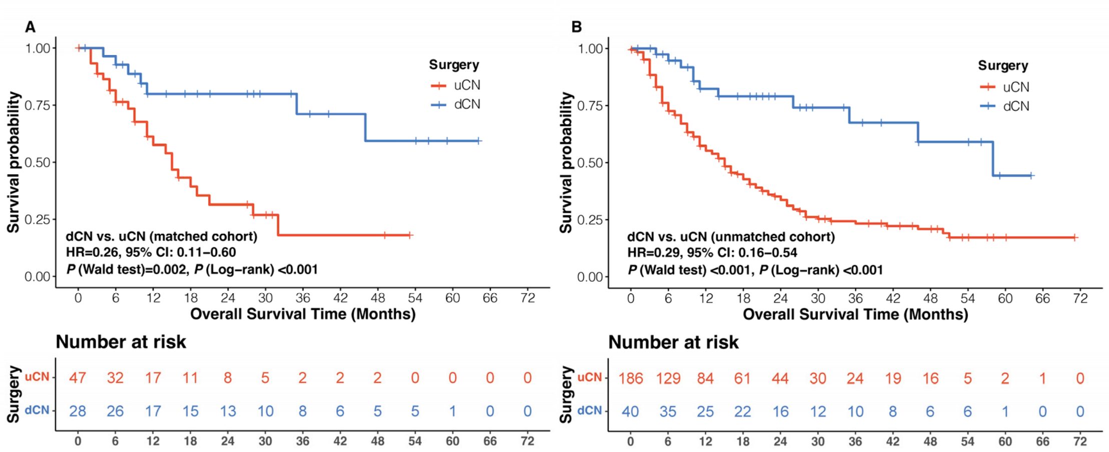
A
Survival probability
1.00
0.75
0.50
0.25
0.00
Surgery
uCN
dCN
dCN vs. uCN (matched cohort)
HR=0.26, 95% CI: 0.11–0.60
P (Wald test)=0.002, P (Log–rank) <0.001
0 6 12 18 24 30 36 42 48 54 60 66 72
Overall Survival Time (Months)
Number at risk
Surgery
uCN 47 32 17 11 8 5 2 2 2 0 0 0 0
dCN 28 26 17 15 13 10 8 6 5 5 1 0 0
0 6 12 18 24 30 36 42 48 54 60 66 72
B
Survival probability
1.00
0.75
0.50
0.25
0.00
Surgery
uCN
dCN
dCN vs. uCN (unmatched cohort)
HR=0.29, 95% CI: 0.16–0.54
P (Wald test) <0.001, P (Log–rank) <0.001
0 6 12 18 24 30 36 42 48 54 60 66 72
Overall Survival Time (Months)
Number at risk
Surgery
uCN 186 129 84 61 44 30 24 19 16 5 2 1 0
dCN 40 35 25 22 16 12 10 8 6 6 1 0 0
0 6 12 18 24 30 36 42 48 54 60 66 72
